# Metabolomics-Based Analysis on the Effect and Metabolic Response of Mycelia by Sawdust Addition from *Hypsizygus marmoreus*

**DOI:** 10.3390/foods13060867

**Published:** 2024-03-13

**Authors:** Jiahuan Li, Jiacheng Xie, Zenan Huang, Peilei Yang, Deng Li, Liding Chen, Shujing Sun

**Affiliations:** 1 College of Life Sciences, Fujian Agriculture and Forestry University, Fuzhou 350002, China; lijiahuansw05@fafu.edu.cn (J.L.); 3200537080@fafu.edu.cn (J.X.); 1200525045@fafu.edu.cn (Z.H.); 5220543021@fafu.edu.cn (P.Y.); 12305014038@fafu.edu.cn (D.L.); chenliding@fafu.edu.cn (L.C.); 2 Gutian Edible Fungi Research Institute, Fujian Agriculture and Forestry University, Ningde 352200, China

**Keywords:** *Hypsizygus marmoreus*, mycelia, sawdust, nutrient composition, metabolomics

## Abstract

The composition of culture substrate is an important environmental factor that affects the growth and metabolism of *Hypsizygus marmoreus*, and sawdust is commonly used as the substrate for cultivating mushrooms. However, the influences of sawdust on metabolic level of *H. marmoreus* in mycelial growth is little reported. In this study, the effect of sawdust addition on mycelial growth rate, morphological characteristics and nutrient content of *H. marmoreus* was explored, and the metabolic response was analyzed based on LC-MS/MS. The results showed the mycelial growth rates and the number of mycelial clamp connections in sawdust medium A and sawdust medium B were significantly higher than that of the basic medium (Control). The mycelial morphology in sawdust medium A was denser, with higher edge trimness and stronger aerial mycelia. The contents of crude fiber, crude protein and polysaccharide of the mycelia from sawdust medium A increased by 85.15%, 90.65% and 92.61%, respectively, compared to that in the basic medium. A total of 551 metabolites were identified and obtained. The differential accumulated metabolites (DAMs) were mainly amino acids, lipids compounds and carbohydrates. It was speculated that the addition of sawdust played a vital role in promoting the cell division and, thus, the formation of clamp connections in *H. marmoreus* mycelia. Regarding amino acids, the metabolism of glycine, serine and ABC transporters was active with the increase in sawdust, thereby increasing the protein content. And some valuable bioactive molecules were found, such as docosahexaenoic acid (DHA). This study will lay the foundation for further research on the substance transformation and quality improvement of cultivation substrate for mushrooms.

## 1. Introduction

*Hypsizygus marmoreus* is an important commercial edible mushroom and widely cultivated in East Asia. It has become one of the most popular edible mushrooms due to its high nutritional value [1]. Mycelium is one of the important stages of mushroom culture, and its culture substrate is another key element because of its essential nutrients for mushroom growth [2]. The composition of the culture substrate plays a vital role in the cultivation of *H. marmoreus*. As the main component of the culture substrate, sawdust can provide abundant carbon sources for the growth and development of *H. marmoreus*. The composition and amount of sawdust have an important effect on the growth rate, mycelia density, yield and quality of the fruiting body of *H. marmoreus* [3,4]. Currently, most research on the cultivation of *H. marmoreus* mainly focuses on the development of new formulations and the improvement of yield and quality, while the related research on the influence of different culture substrates on the expression of metabolites and the changes in metabolic pathways of *H. marmoreus* is relatively little. *H. marmoreus* is worth studying as a source of indole compounds and other bioactive substances with health-promoting activities [5].

Metabolomics is a high-throughput method to analyze many endogenous metabolites in complex biological system [6,7]; it can explain the metabolic network and internal metabolism of organisms under specific conditions [8], and metabolomics has been widely used in many research fields such as animals [9,10,11], plants [12,13] and microorganisms [14,15,16,17]. Currently, metabolomics has also been applied in the field of edible fungi, mainly focusing on the difference analysis of metabolites between wild cultivars and main cultivars to identify the quality of edible fungi [18] and construction of metabolic profiles of different tissue parts of edible fungi [19]. Therefore, metabolomics technology can be used to deeply understand the metabolic response of edible fungi to different culture substrates. The relationship between metabolic products and the physiological and nutritional properties can be better understood by analyzing the changes in the metabolites of edible fungi under different culture substrates, so that the formula of the cultivation substrate can be improved to promote the nutritional quality and medicinal value of the edible fungi.

The mycelia of *H. marmoreus* cultured with different proportions of sawdust instead of glucose as the main carbon source were used as the research materials. Liquid chromatography tandem mass spectrometry (LC-MS/MS), combined with multiple statistical analyses, was used to investigate the metabolome profiles of *H. marmoreus* mycelia and explore the mechanism of the metabolic difference of *H. marmoreus* mycelia under the different additions of sawdust. These results would help us to deepen our understanding of the biochemical mechanism behind the decomposition and utilization of sawdust and nutrient transformation of *H. marmoreus* from the perspective of metabolism, and also provide a theoretical basis for exploring and improving the culture formula of *H. marmoreus* and the use of mycelia as the source of active substances of edible fungi.

## 2. Materials and Methods

### 2.1. Strains and Culture Conditions

A pure culture of *H. marmoreus* called ‘Minzhen 2’, which was bred by Fujian Agriculture and Forestry University, Xinluo Huohuo Edible Mushroom Co., Ltd., Longyan, China and Taiheqiang Agricultural Development Co., Ltd., Longyan, China, was used in this study.

The strain was incubated in three types of media at 25 °C. The basic medium (Control) comprised 20 g glucose, 2 g tryptone, 1 g MgSO_4_·7H_2_O, 0.46 g KH_2_PO_4_, 1 g K_2_HPO_4_, 20 g agar and 0.1 g vitamin B1 (VB_1_) in 1 L water, pH normal. The sawdust media A and B were, respectively, 5 g and 15 g of sawdust (manual crushing broad-leaved trees mixtures), instead of the corresponding amount of glucose. All chemical reagents were purchased from Sangon Biotech Co., Ltd., Shanghai, China

### 2.2. Mycelial Growth Rate and Morphology Analysis

The mycelial discs (d = 1 cm) were incubated in three types of media under sterile conditions, with six replicates for each medium. The mycelial growth rate was measured and calculated on the 5th, 7th and 10th days. The morphological characteristics of mycelia were observed when they grew to about 2/3 of the plate.

Additionally, three sterile coverslips were inserted equidistantly into the media at the edge of the mycelia to separate the aerial mycelia from the media when the mycelia grew to 1/3 of the plate. The coverslips were carefully removed from the plate after the aerial mycelia climbed onto the coverslips, the microscopic structure of the edge mycelia was observed, and the number of clamp connections in a visual field was calculated. The number of mycelial clamp connections was calculated by dividing the number of all clamp connections in the selected observation field by the number of mycelial branches with clamp connections. The mycelia from 10 days of culture were taken, glutaraldehyde-fixed for 8 h, washed with different gradients of ethanol and then fixed with isoamyl acetate for 2 h and stored at −80 °C overnight. The samples were dried by freeze dryer (Christ Alpha 2-4 LSCbasic, Osterode, Germany), and the mycelial ultrastructure was observed by scanning electron microscope (ZEISS ULTRA 55 Oberkochen, Germany).

### 2.3. Nutrient Content Analysis

The polysaccharide of the mycelia was determined by phenol-sulfuric acid method with reference to NY/T1676-2008 [20]. The crude protein was conducted using Kjeldahl method with reference to GB 5009.5-2016 [21]. The Crude fiber determination was improved with reference to GB/T 5009.10-2003 [22].

### 2.4. Samples Preparation

Under sterile conditions, the mycelia cultured in a plate for 15 days were scraped and placed in a 2 mL sterilized centrifuge tube, sealed with parafilm and stored at −80 °C for further experiments.

### 2.5. Metabolite Extraction and UHPLC-MS/MS Conditions

Mycelial samples of each culture condition were milled and weighed for 60 mg accurately, then transferred into 2 mL centrifuge tubes. The 500 µL of methanol (pre-cooled at −20 °C) and 500 µL H_2_O (4 °C) were added into the centrifuge tube and vortex for 30 s, then 100 mg glass beads were added. The tubes were placed into liquid nitrogen for 5 min and thawed at room temperature, and then the tubes were put into the tissue grinder and ground for 2 min at 55 Hz; the step above was repeated twice. The tubes were centrifuged at 4 °C for 10 min at 12,000 rpm. Next, the supernatants were transferred into new centrifuge tubes and concentrated to dryness in a vacuum. The samples were dissolved with 300 µL 2-chlorobenzalanine (4 ppm) methanol aqueous solution (1:1, 4 °C), and the supernatants were filtered through 0.22 µm membrane to obtain the prepared samples for LC-MS. Of each sample, 20 µL was taken to the quality control (QC) samples, and the rest were used for LC-MS detection.

For the chromatographic study, the Thermo Vanquish UHPLC instrument was used with an ACQUITY UPLC^®^ HSS T3 (150 × 2.1 mm, 1.8 µm, Waters) column maintained at 40 °C. The temperature of the autosampler was 8 °C. Gradient elution of analytes was carried out with 0.1% formic acid in water (C) and 0.1% formic acid in acetonitrile (D) or 5 mM ammonium formate in water (A) and acetonitrile (B) at a flow rate of 0.25 mL/min. Injection of 2 μL of each sample was carried out after equilibration. An increasing linear gradient of solvent B (*v*/*v*) was used as follows: 0~1 min, 2% B/D; 1~9 min, 2%~50% B/D; 9~12 min, 50%~98% B/D; 12~13.5 min, 98% B/D; 13.5~14 min, 98%~2% B/D; 14~20 min, 2% D-positive model (14~17 min, 2% B-negative model) [23].

The Thermo QE-HF-X instrument was used for the mass spectrometry study with electrospray ionization (ESI) and cation–anion ionization mode. The spray voltages for positive and negative modes were 3.5 kV and 2.5 kV, respectively. Sheath gas and auxiliary gas were set at 30 and 10 arbitrary units, respectively. The capillary temperature was 325 °C. The analyzer scanned over a mass range of *m*/*z* 81-1000 for a full scan at a mass resolution of 60,000. Data-dependent acquisition (DDA) MS/MS experiments were performed with an HCD scan. The normalized collision energy was 30 eV. Dynamic exclusion was implemented to remove some unnecessary information in MS/MS spectr [24].

### 2.6. Metabolome Data Analysis

The metabolites identification of the raw data was first confirmed with the exact molecular weight (molecular weight error < 30 ppm) and was further matched with annotations to obtain accurate information on metabolites, according to the fragmentation information obtained by MS/MS mode in the Human Metabolome Database (HMDB) (http://www.hmdb.ca (accessed on 7 January 2022)), Metlin (http://metlin.scripps.edu (accessed on 4 February 2021)), massbank (http://www.massbank.jp/ (accessed on 11 April 2023)), LipidMaps (http://www.lipidmaps.org (accessed on 1 January 2003)) and mzclound (https://www.mzcloud.org (accessed on 12 December 2013)).

The SIMCA-P(v13.0) and R-ropls packages were used for all multivariate data analyses and modeling [25]. Data were standardized using autoscaling. The multivariate statistical analysis methods used in this article, including Principal Component Analysis (PCA) and Partial Least Squares-Discriminate Analysis (PLS-DA), were applied to reveal the distinctions in the metabolites between varied comparison groups. The differentially accumulated metabolites (DAMs) were screened according to the following two criteria: variable importance in projection (VIP) values > 1.00 for the first principal component of the OPLS-DA model and *p*-value < 0.05 for the *t*-test. Then, hierarchical clustering analysis was performed for DAMs among different sample groups.

The connection among DAMs for constructing metabolic pathways was analyzed based on databases including KEGG (http://www.genome.jp/kegg/ (accessed on 15 May 2023)) and MetaboAnalyst (https://www.metaboanalyst.ca/ (accessed on 17 June 2022)) [26].

### 2.7. Statistical Analysis

All of the statistical analyses were performed by IBM SPSS Statistics 20 software. The significant differences among samples from different treatments were assessed using one-way ANOVA with the Tukey post hoc comparison test.

## 3. Results

### 3.1. Effect of Sawdust Addition on Mycelial Growth Rate and Morphological Characteristics of H. marmoreus

**Figure 1:**
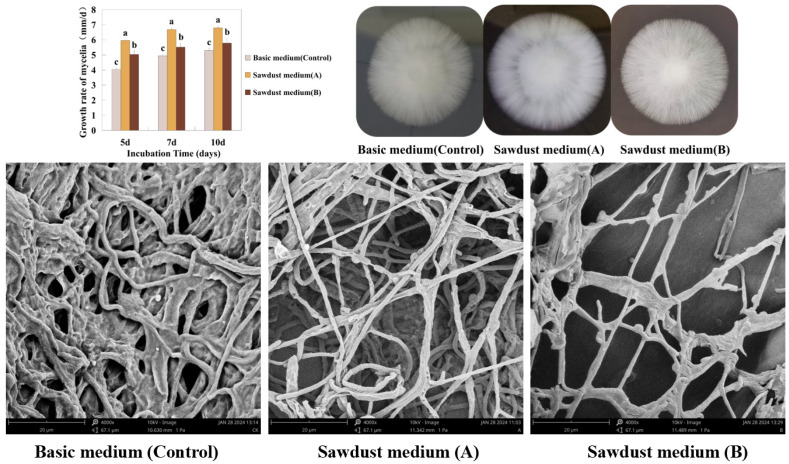
Phenotypic analysis of *H. marmoreus* mycelia cultured in three types of medium. (**A**) The mycelial growth rate of *H. marmoreus*. The differences among the three groups were represented by different lower-case letters as tested by Tukey’s HSD test (*p* < 0.05). (**B**) The mycelial morphology of *H. marmoreus*. (**C**) The differences in the number of clamp connections among each treatment by microscopic observations (4000×).

The mycelial growth rates in basic medium (Control), sawdust medium A and sawdust medium B for 10 days were 5.30 ± 0.17 mm/d, 6.79 ± 0.14 mm/d and 5.79 ± 0.30 mm/d, respectively. The mycelial growth rates in sawdust medium A and sawdust medium B were significantly higher than that of the control (Figure 1A, *p* < 0.05). The mycelial morphology in sawdust medium A was denser, with higher edge trimness and stronger aerial mycelia (Table 1 and Figure 1B). The number of clamp connections of each mycelium in the three different conditions (basic medium, sawdust medium A, sawdust medium B) was 1.36 ± 0.18, 1.65 ± 0.3, 1.92 ± 0.24 (Figure 1C, *p* < 0.05). The highest number of mycelial clamp connections was observed in sawdust medium B. The number of mycelial clamp connections increased by 41.2% and 16.4% compared to those in basic medium and sawdust medium A, respectively.

### 3.2. Influence of Sawdust Addition on Nutrient Contents of H. marmoreus

The mycelia cultivated in sawdust medium A had the highest crude fiber, crude protein and polysaccharide contents of 6.91 g/g, 70.94 g/100 g and 4.43 g/100 g, respectively (Table 1). The contents of crude fiber, crude protein and polysaccharide increased by 85.15%, 90.65% and 92.61% compared to that in basic medium (Control), respectively. The addition of sawdust was conducive to the accumulation of mycelial crude fiber, polysaccharide and crude protein nutrients, especially in a certain amount of fast-acting carbon source glucose based on the addition of sawdust.

### 3.3. Metabolite Characterization and Classification

**Figure 2:**
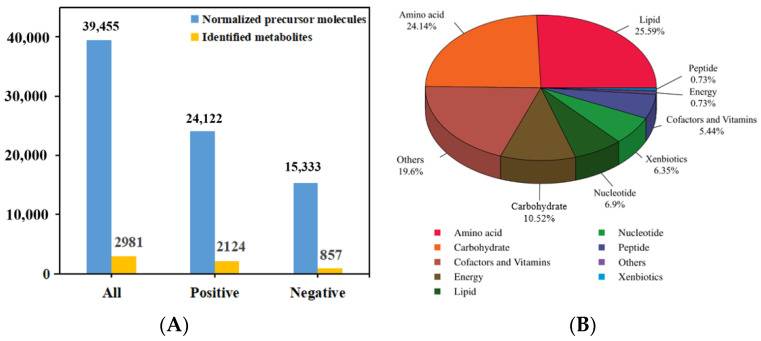
Classification of the identified metabolomics data of *H. marmoreus* mycelia. (**A**) Histogram of normalized precursor molecules and metabolites. (**B**) Classification pie chart of 551 metabolites for secondary identification.

Three sample groups with six biological replicates were performed, and the total ion chromatogram (TIC) in positive and negative ion modes was shown in Figure S1. After normalizing the raw data, the LC-MS analysis of mycelia under different culture conditions yielded 24,122 and 15,333 precursor molecules in the positive and negative ion modes, respectively. A total of 2981 metabolites were obtained after identification, of which 2124 and 857 were identified in positive and negative ion modes, respectively (Figure 2A). After secondary identification, a total of 551 metabolites were obtained. These metabolites could be categorized into 9 classes, mainly including lipids (25.59%), amino acids (24.14%), carbohydrates (10.53%), nucleotides (6.9%), cofactors and vitamins (5.44%) (Figure 2B), which showed that the metabolites of the mycelia had the largest proportion of lipid and amino acid substances. Interestingly, the metabolites contained some peptides and xenbiotics, which may be derived from the secondary metabolism of *Hypsizygus marmoreus* mycelium using amino acids, lipid and carbohydrates as growth substrates.

### 3.4. Multivariate Statistical Analysis of Metabolites

**Figure 3:**
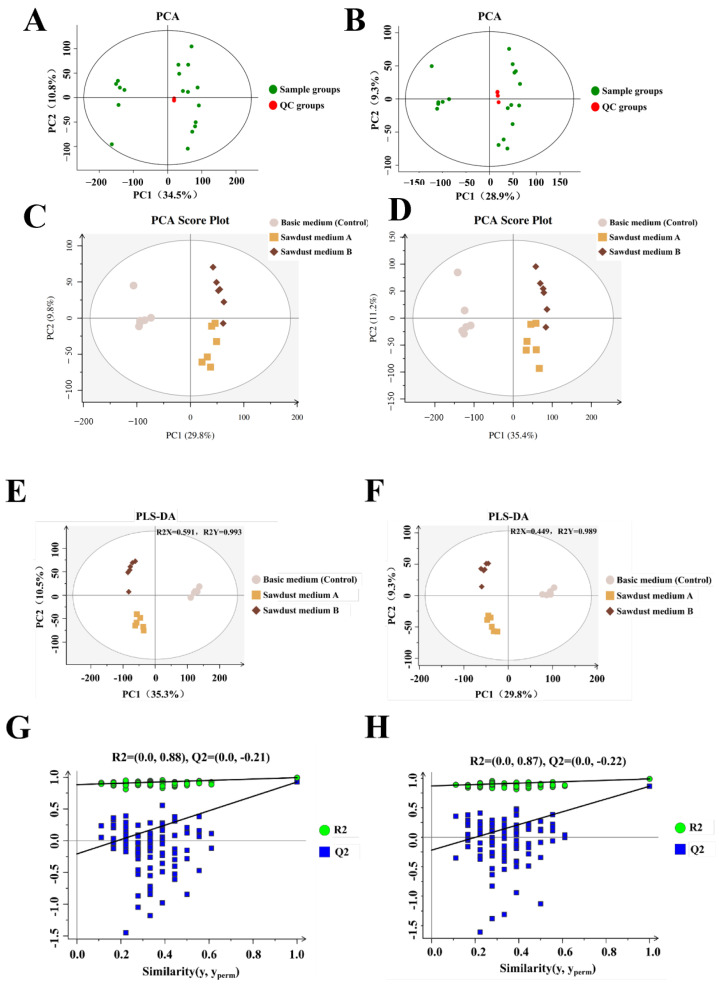
Multivariate analysis and permutation test of LC-MS-based metabolomics data of *H. marmoreus* mycelia. (**A**,**B**) Principal component analysis (PCA) of *H. marmoreus* metabolome of sample groups and quality control (QC) group in the positive and negative ion modes. (**C**,**D**) Principal component analysis (PCA) of *H. marmoreus* metabolome of three sample groups in positive and negative ion modes. (**E**,**F**) Partial least squares-discriminate analysis (PLS-DA) in the positive and negative ion modes, respectively. (**G**,**H**) Permutation test results of the PLS-DA mode in the positive and negative ion modes, respectively.

To characterize the overall metabolites differences among the three sample groups and QC group, the principal component analysis (PCA) was performed. The results showed that the QC samples were densely distributed in the middle of the score plot, which suggested that the stability of the metabolomics platform was fine. The detection data were reliable throughout the operation (Figure 3A,B). Both positive and negative ion models of PCA score plots among the three sample groups were presented in Figure 3C,D. The first two principal components from eluents C and D, PC1 and PC2, explained 35.4% and 11.2% of the total variation in the positive ion mode and 29.8% and 9.8% of the total variation in the negative ion mode, respectively. The PCA results showed that the metabolites among the three sample groups were distinctly separated from each other and PC1 values proved that the larger metabolite changes happened in mycelia.

To minimize intra-group errors and eliminate random errors unrelated to the research purpose as much as possible, the supervised partial least squares data analysis (PLS-DA) model was carried out to analyze the differences among mycelia cultured under three different conditions. As shown in Figure 3E,F, the same separations were also found in the PLS-DA model. Furthermore, to identify the accuracy of the metabolomics data, the cross-validation and permutation test for PLS-DA was applied to assess the goodness of fit of the PLS-DA models. The R^2^X (cum) and R^2^Y (cum) of the PLS-DA models were 0.591 (+)/0.449 (−) and 0.993 (+)/0.989 (−), respectively, which indicated the good predictability and interpretability of the PLS-DA models. The permutation test plots showed that all the test Q^2^ points were lower than the original Q^2^ point (Figure 3G,H), and the intersection of the regression line at Q^2^ points with the ordinate were −0.21 (+) and −0.22 (−), which were both less than zero and firmly suggested that the PLS-DA models were not overfitted. In general, the results showed that the metabolic status of mycelia changed distinctly under different amounts of sawdust addition.

### 3.5. DAMs Screening and Analysis

**Figure 4:**
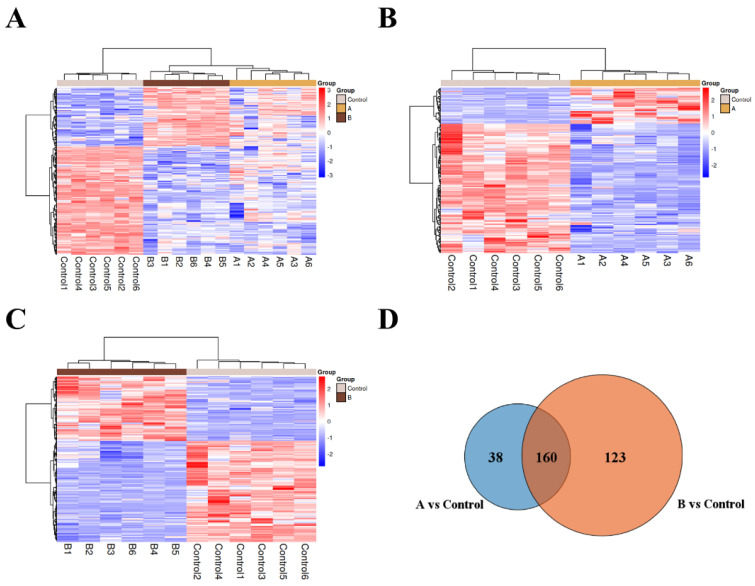
Differentially accumulated metabolites (DAMs) among *H. marmoreus* mycelia under different culture conditions. (**A**) Heatmap of the DAMs in Control vs. A vs. B. (**B**) Heatmap of the DAMs in Control vs. A. (**C**) Heatmap of the DAMs in Control vs. B. (**D**) Venn diagram of DAMs identified from the two comparisons of *H. marmoreus* mycelia (A vs. Control and B vs. Control). The numbers of common and unique DAMs between each comparison group were shown in the Venn diagram.

The differentially accumulated metabolites (DAMs) were further screened according to the principle of VIP values > 1.00 for the first principal component of the OPLS-DA model and *p* value < 0.05 for the *t*-test [27], and a total of 346 DAMs were identified by pairwise comparison between sawdust medium A (15 g sawdust replacement), B (20 g sawdust replacement) and Control (basic medium). Then, the hierarchical cluster analysis heat map was drawn based on the relative quantitative values of 236 DAMs obtained by comparison and differential metabolite screening among the three groups. As shown in Figure 4A, the metabolic information of the same treatment group can be clustered together, showing that the screened DAMs can accurately reflect the metabolic information of each treatment group. The screened DAMs can also be clustered in a branch, revealing that the metabolites of each cluster may involve the same metabolic pathway or have similar functional characteristics.

A total of 198 DAMs were detected between group A and Control. The major DAMs in A vs. Control could be categorized into five classes, including lipids (25.25%), amino acids (24.24%), carbohydrates (9.6%), cofactors and vitamins (5.56%) and nucleotides (5.56%) (Table S1). Among them, lipids and amino acids accounted for a large proportion. The lipids could be further classified as 11 fatty acyls, 7 prenol lipids, 5 steroids and steroid derivatives, 2 azacyclic compounds, 2 benzene and substituted derivatives, 2 carboxylic acids and derivatives, 2 flavonoids, 2 organonitrogen compounds, 2 organooxygen compounds, 1 keto acids and derivatives, 1 linear 1,3-diarylpropanoids, 1 hydroxy acid and derivatives, 1 stilbene and 11 other lipids. The amino acids could also be further categorized as 24 carboxylic acids and derivatives, 4 benzene and substituted derivatives, 4 indoles and derivatives, 2 azacyclic compounds, 2 cinnamic acids and derivatives, 1 carboximidic acid and derivatives, 1 endogenous metabolite, 1 organooxygen compound, 1 fatty acyls, 1 organic sulfonic acids and derivatives and 7 other amino acids. In addition, there were 43 up-regulated metabolites in A, mainly composed of 18.6% amino acids and 34.9% lipids, and 155 down-regulated metabolites in A, which consisted mainly of 25.8% amino acids and 22.6% lipids (Figure 4B and Table S2). Furthermore, among the 198 DAMs obtained in A vs. Control, the up-regulation of differential amino acids accounted for 16.7% of the total differential amino acids. The up-regulation of differential lipids accounted for 30% of the total lipids, which means that the overall richness of amino acids and lipids in *H. marmoreus* mycelia decreased under A treatment.

The majority of DAMs in B vs. Control could also be divided into five categories, consisting of lipids (27.21%), amino acids (22.97%), carbohydrates (10.25%), nucleotides (7.07%) and cofactors and vitamins (6.01%) (Table S1). Seventy-seven lipids contained 21 fatty acyls, 9 prenol lipids, 8 steroids and steroid derivatives, 5 carboxylic acids and derivatives, 4 azacyclic compounds, 4 flavonoids, 3 organonitrogen compounds, 2 organooxygen compounds, 1 benzene and substituted derivatives, 1 keto acids and derivatives, 1 hydroxy acids and derivatives, 1 pyran, 1 linear 1,3-diarylpropanoids, 1 glycerophospholipid and 15 other lipids. Likewise, sixty-five amino acids were further classified as 33 carboxylic acids and derivatives, 4 benzene and substituted derivatives, 4 indoles and derivatives, 2 cinnamic acids and derivatives, 2 5′-deoxyribonucleosides, 2 azacyclic compounds and 18 other amino acids. Of the 283 DAMs obtained by comparing group B and Control, 110 metabolites were up-regulated, and 173 were down-regulated in B. Among 110 up-regulated metabolites, amino acids accounted for 20.9%, and lipids accounted for 28.2%. In the same way, among 173 down-regulated metabolites, 24.3% were amino acids, and 26.6% were lipids (Figure 4C and Table S3).

Multiple comparisons showed that 160 DAMs were detected in both A vs. Control and B vs. Control, among which the lipids and amino acids accounted for the majority, making up 28.13% and 20.63% of the 160 DAMs, respectively (Figure 4D and Table S4). Additionally, 123 DAMs were unique in group B vs. Control, and 38 DAMs were unique in group A vs. Control.

All the above results indicated that the addition of sawdust mainly affected the metabolism of lipids and amino acids in mycelia. Moreover, most of lipids and amino acids were down-regulated.

### 3.6. DAAs and DALs in B vs. Control

**Figure 5:**
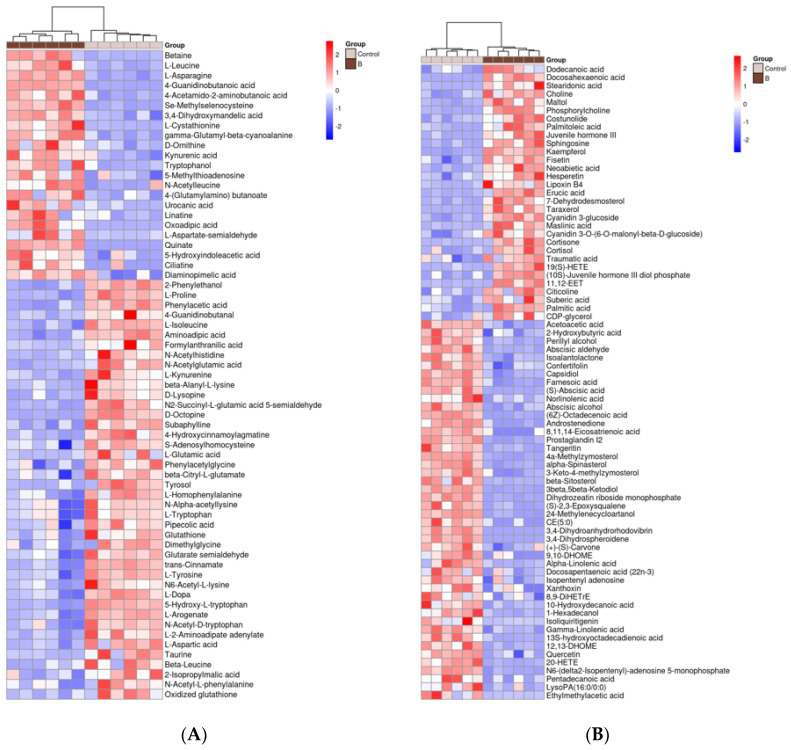
Differentially accumulated amino acids (DAAs) and differentially accumulated lipids (DALs) in B vs. Control. (**A**) Heatmap of the DAAs. (**B**) Heatmap of the DALs.

The differentially accumulated amino acids (DAAs) and differentially accumulated lipids (DALs) in the DAMs screening results of B vs. Control were selected for further elaboration as follows. Focusing on the 65 DAAs and 77 DALs between group B and Control (Figure 5 and Tables S6 and S7), the up-regulated DAAs and DALs accounted for 35.4% and 40.3% of the total DAAs and DALs in B vs. Control, respectively. Conversely, the down-regulated DAAs and DALs accounted for more than half of the total DAAs and DALs.

In detail, it was found that twenty-three amino acids up-regulated (FC > 1) in group B mainly included oxoadipic acid (VIP = 1.41, log_2_FC = 21.00), L-Cystathionine (VIP = 1.28, log_2_FC = 4.23), quinate (VIP = 1.61, log_2_FC = 3.62), L-Aspartate-semialdehyde (VIP = 1.09, log_2_FC = 3.52), 4-Guanidinobutanoic acid (VIP = 1.51, log_2_FC = 3.07), etc. Forty-two amino acids were down-regulated (FC < 1), mainly including L-Glutamic acid (VIP = 1.08, log_2_FC = −3.38), taurine (VIP = 1.22, log_2_FC = −3.36), D-Octopine (VIP = 1.47, log_2_FC = −2.91), N-Acetylhistidine (VIP = 1.16, log_2_FC = −2.56), formylanthranilic acid (VIP = 1.00, log_2_FC = −2.55), etc.

Among the thirty-one up-regulated lipids in B, the top five lipids with the largest fold change were docosahexaenoic acid (VIP = 1.42, log_2_FC = 19.75), stearidonic acid (VIP = 1.24, log_2_FC = 5.46), taraxerol (VIP = 1.34, log_2_FC = 5.18), cortisone (VIP = 1.22, log_2_FC = 4.72) and erucic acid (VIP = 1.31, log_2_FC = 4.35). And there were forty-six down-regulated lipids, mainly including 3,4-Dihydrospheroidene (VIP = 1.48, log_2_FC = −7.17), (S)-Abscisic acid (VIP = 1.45, log_2_FC = −5.92), 3,4-Dihydroanhydrorhodovibrin (VIP = 1.47, log_2_FC = −5.36), beta-Sitosterol (VIP = 1.31, log_2_FC = −5.06), prostaglandin I2 (VIP = 1.49, log_2_FC = −4.34), etc.

In addition, among the DAMs between group B and Control, we found some bioactive molecules with large accumulation and great utilization value. Here, we listed only a few of them, such as docosahexaenoic acid (DHA) (VIP = 1.42, log_2_FC = 19.75), mitomycin (VIP = 1.48, log_2_FC = 7.61), taraxerol (VIP = 1.34, log_2_FC = 5.18), maslinic acid (VIP = 1.20, log_2_FC = 3.35).

### 3.7. The KEGG Analysis of DAMs

**Figure 6:**
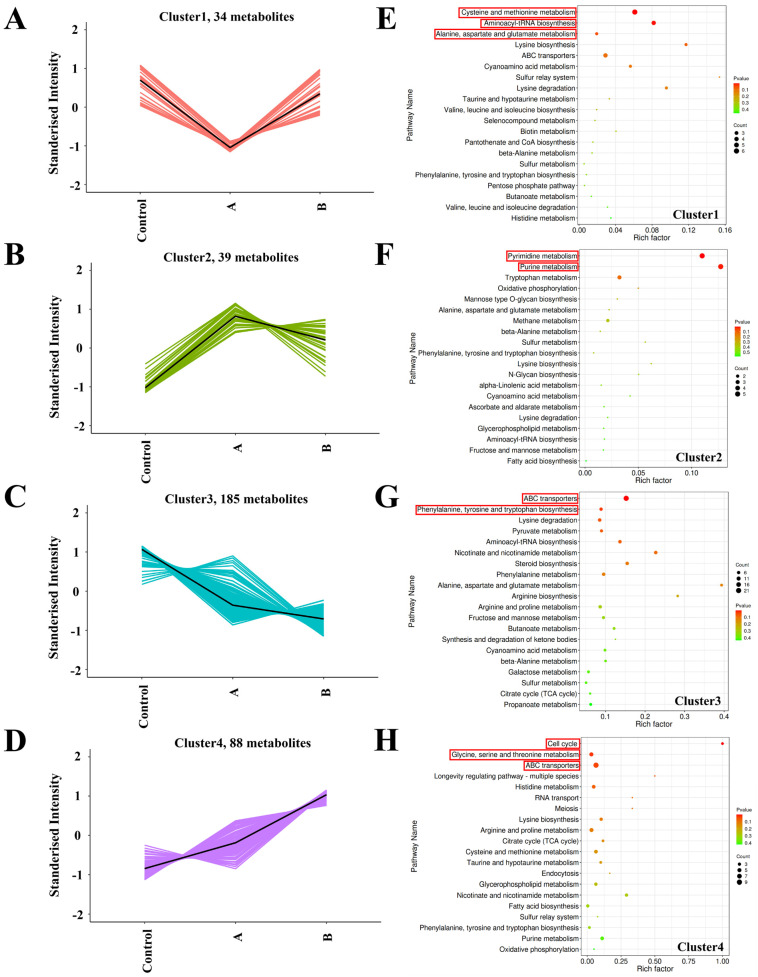
The K-means clustering and KEGG enrichment analyses are based on differentially accumulated metabolites (DAMs) within each cluster from samples A (5 g of sawdust) and B (15 g of sawdust). (**A**–**D**) The K-means clustering groups of the DAMs expression profiles of *H. marmoreus* mycelia cultured under three treatments. The y-axis represents the standardized amount of each metabolite, and the x-axis represents the different treatment groups. (**E**–**H**) Top 20 enriched KEGG pathways of the DAMs gathered in the corresponding cluster. The color intensity (green to red) is proportional to the enrichment significance, and the circle size indicates the number of enriched metabolites. The pathways with significant differences were highlighted in a red box (*p* < 0.05).

First, K-means clustering analysis was performed on 346 DAMs. All 346 DAMs were clustered according to the trend of their relative expression abundance in the three treatment groups, and four clusters were obtained (Figure 6A–D and Table S5). The 34, 39, 185 and 88 DAMs converged in cluster 1, cluster 2, cluster 3 and cluster 4, and fit these four trends. To further explore the mechanism behind the effect of different amounts of sawdust addition on the metabolism of *H. marmoreus* mycelia, annotation and functional enrichment analysis was conducted on the DAMs gathered in the four clusters based on the KEGG database, and the corresponding top 20 enriched metabolic pathways were shown in Figure 6E–H.

In cluster 1, the accumulation of DAMs was higher in the Control group and B group but lower in the A group (Figure 6A). The 39 DAMs in cluster 1 were associated with 26 KEGG pathways, and half of the top 20 enriched pathways were mainly associated with amino acid metabolism. Among them, “Cysteine and methionine metabolism” (ko00270, enriched metabolite count = 6), “Aminoacyl-tRNA biosynthesis” (ko00970, enriched metabolite count = 4) and “Alanine, aspartate and glutamate metabolism” (ko00250, enriched metabolite count = 2) were the significantly enriched pathways (*p* < 0.05) (Figure 6E). The 34 DAMs in cluster 2 were distributed in 24 KEGG pathways, and in the top 20 enriched pathways were mainly related to amino acid metabolism, lipid metabolism, carbohydrate metabolism and energy metabolism, among which “pyrimidine metabolism” (ko00240, enriched metabolite count = 5) and “purine metabolism” (ko00230, enriched metabolite count = 5) were the significant enrichment pathways (*p* < 0.05) (Figure 6F). In addition, the expression trend of metabolites in different treatment groups exhibited an opposed result with that in cluster 1 and was consistent with the trend of mycelial growth rate (Figure 6B), which indicated that “pyrimidine metabolism” and “purine metabolism” pathways were correlated with mycelia’s growth and development.

The 185 DAMs from cluster 3 were associated with 59 KEGG pathways, most of which were associated with amino acid metabolism (including 14 pathways), carbohydrate metabolism (including 13 pathways) and metabolism of cofactors and vitamins (including 9 pathways). Moreover, “ABC transporters” (ko02010, enriched metabolite count = 21) and “phenylalanine, tyrosine and tryptophan biosynthesis” (ko00400, enriched metabolite count = 6) were the significant enrichment pathways in cluster 3 (*p* < 0.05) (Figure 6G). The 88 DAMs in cluster 4 were enriched to 56 KEGG pathways, and the majority were related to amino acid metabolism (including 13 pathways), carbohydrate metabolism (including 11 pathways) and lipid metabolism (including 9 pathways). The significantly enriched pathways were “Cell cycle” (ko04111, enriched metabolite count = 2), “Glycine, serine and threonine metabolism” (ko00260, enriched metabolite count = 5) and “ABC transporters” (ko02010, enriched metabolite count = 9) (*p* < 0.05) (Figure 6H). Additionally, it was noteworthy that the variation trend of DAMs in cluster 4 was consistent with the content of sawdust in the medium, and the number of clamp connections in each group was also consistent. It was speculated that the addition of sawdust played a vital role in promoting the cell division and the formation of clamp connections in *H. marmoreus* mycelia, which was also confirmed by the significantly enriched “cell cycle” pathway in KEGG of cluster 4. Regarding amino acids, the metabolism of glycine, serine and threonine was active with the increase in sawdust. At the same time, the synthesis of phenylalanine, tyrosine and tryptophan was the opposite. In addition, “ABC transporters” was the significantly enriched pathway in both cluster 3 and 4.

## 4. Discussion

The expression and accumulation of metabolites is a complex process in vivo and mainly determined by external environmental factors, heredity and developmental processes [28,29,30]. According to the phenotypic results of mycelia, there were significant differences between mycelia cultured in different sawdust supplemental levels, which meant that the metabolic characteristics of mycelia in different groups also changed. Some studies have also shown that using sawdust as a carbon source to cultivate mushrooms greatly improves mycelia’s growth rate and the content of nutrients in fruiting bodies [31,32].The results suggested that the properties of *H. marmoreus* cultured in sawdust medium A were markedly better than those cultured in basic medium (Control) and sawdust medium B, which indicated some changes in substrate absorption, utilization and metabolic transformation had taken place in the mycelia of *H. marmoreus*.

The amino acids and lipids account for the largest proportion of DAMs. The trend of DAMs is consistent with the trend of mycelial phenotype and nutrient composition, in which the most significant enrichment pathways are pyrimidine metabolism and purine metabolism. Purine and pyrimidine nucleotides are important components of nucleic acid synthesis and are involved in a number of biochemical processes, and their biosynthesis and metabolism are essential for the growth and development of organisms [33]. Some studies on nucleotide metabolism in fungi have shown that purine nucleotide metabolism is specifically regulated in the sexual reproduction of mycelium [34], and that mycelium promotes ab initio synthesis and exuberant metabolism of purine nucleotides under rapid growth conditions. Meanwhile, some valuable bioactive molecules were also found in the up-regulated DAMs, such as docosahexaenoic acid (DHA), whose log_2_FC reached 19.75 in group B compared with control. DHA has functions related to improving health, such as altering cell membrane structure, cell protein function, lipid mediators production, and gene expression patterns, and DHA is essential for correct visual and neurological development, making mushrooms containing DHA suitable as nutritional supplements for pregnant women and newborn [35,36,37,38]. Taraxerol, mitomycin and maslinic acid have some research value in relieving acute inflammation and anti-tumor effects [39,40,41]. Due to the limitations of untargeted metabolomics, the quantitative values of various bioactive molecules detected in *H. marmoreus* mycelia cultured with sawdust addition must be further verified. The significant up-regulation and increase in metabolite species corresponded to a significant rise in the use of sawdust as a nutrient in the medium, enriching and strengthening multiple energy metabolic pathways of the mycelia. In addition, ABC transporters are part of membrane transport, and their main function involves the active transport of small molecules [42,43]. We speculated that ABC transporters were involved in substance transport during the metabolism and transformation of nutrients such as sawdust and glucose in culture substrates. Microbe-based studies in xylose catabolism have identified multiple specific transporter proteins in the ABC transporter protein family [44], which explains the metabolic up-regulation of ABC transporter proteins, where different carbon sources require different specific ABC transporter proteins for transport to different metabolic pathways.

## 5. Conclusions

In this study, we investigated the effects and correlations of different levels of sawdust addition on the metabolic and phenotypic characteristics of *H. marmoreus* by using non-targeted LC-MS/MS metabolomics combined with phenotypic analysis. The cell cycle pathway was positively correlated with the formation of clamp connections in mushrooms. Analysis of the metabolic pathways that were significantly up-regulated following the replacement of glucose by sawdust mainly included the enhancement of cell cycle metabolism induced by the use of sawdust as a medium, which promoted an increase in the number of mycelial clamp connections. The addition of sawdust addition activated the glycine, serine and threonine metabolism pathway. Still, the phenylalanine, tyrosine and tryptophan biosynthesis pathway were the other way around. This study may provide some valuable resources and references for further research on *H. marmoreus* and would help in future cultivation and quality improvement. Future studies should explore, in depth, the mechanism by which sawdust addition promotes cell metabolism and clamp connections formation.

## Figures and Tables

**Table 1:** Evaluation of the mycelia morphology and nutrient composition of *H. marmoreus* (Average ± SD).

Medium Types	Morphological Characteristics of Mycelia							
	Density	Sturdiness	Whiteness	Edge Tidiness of Colony	Growth Vigor of Aerial Mycelia	Crude Fiber (g/g)	Crude Protein (g/100 g)	Polysaccharide (g/100 g)
Basic medium (Control)	+++	++++	+++	+++	+++	3.57 ± 0.46 ^a^	37.21 ± 1.79 ^c^	2.30 ± 0.21 ^c^
Sawdust medium (A)	++++	++++	++++	++++	++++	6.91 ± 2.44 ^a^	70.94 ± 5.83 ^a^	4.43 ± 0.04 ^a^
Sawdust medium (B)	+++	+++	+++	++++	+++	5.70 ± 0.15 ^a^	50.25 ± 2.30 ^b^	3.96 ± 0.14 ^b^

Notes: ++++: high; +++: moderate; the letters abc represent a difference in significance between the different comparison groups.

## Data Availability

The data presented in this study are available on request from the corresponding author. The raw/processed data required to reproduce these findings cannot be shared at this time as the data also form part of an ongoing study.

## Supplementary Materials

The following supporting information can be downloaded at: https://www.mdpi.com/article/10.3390/foods13060867/s1, Figure S1: Superimposed TIC chromatographic curves of six biological replicates in three groups (Group Control, A and B) in positive (A–C) and negative (D–F) ion modes, respectively. Table S1: Statistics of differentially accumulated metabolites in *H. marmoreus* mycelia. Table S2: List of DAMs between A and Control. Table S3: List of DAMs between B and Control. Table S4: List of DAMs detected in both A vs. Control and B vs. Control. Table S5: List of DAMs in each cluster obtained by K-means clustering analysis. Table S6: List of DAAs between B and Control. Table S7: List of DALs between B and Control.
