# Supplementary material for: Metabolomics-Based Analysis on the Effect and Metabolic Response of Mycelia by Sawdust Addition from Hypsizygus marmoreus
Source: Foods. 2024 Mar 13;13(6):867. doi: 10.3390/foods13060867 (PMC10969604; doi:10.3390/foods13060867)
Supplement: Supplementary file 1 [file foods-13-00867-s001.zip › Supplemental materials-2.pdf]

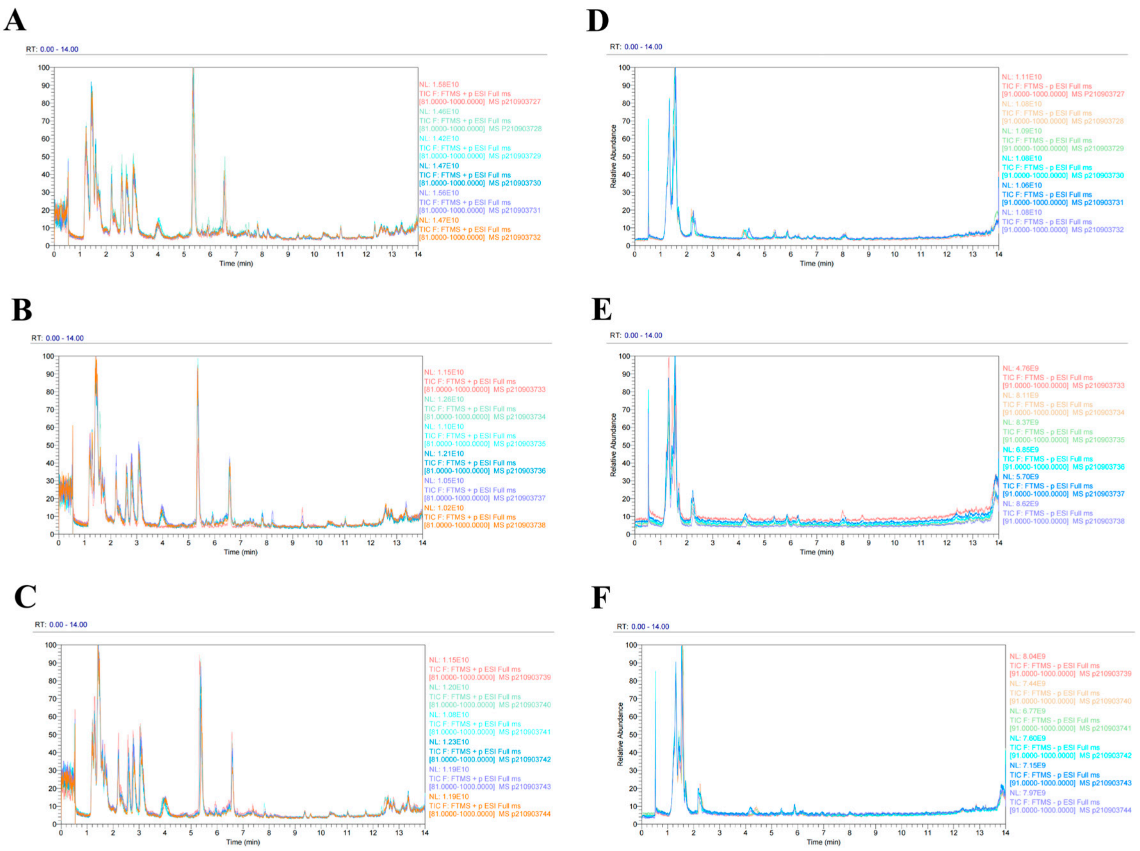

Figure S1. Superimposed TIC chromatographic curves of six biological replicates in three groups (Group Control, A and B) in positive (A-C) and negative (D-F) ion modes, respectively.
